# Supplementary material for: The Patho-Neurophysiological Basis and Treatment of Focal Laryngeal Dystonia: A Narrative Review and Two Case Reports Applying TMS over the Laryngeal Motor Cortex
Source: J Clin Med. 2022 Jun 15;11(12):3453. doi: 10.3390/jcm11123453 (PMC9224879; doi:10.3390/jcm11123453)
Supplement: Supplementary file 1 [file jcm-11-03453-s001.zip › Table S1.pdf]

Table S1: Laryngeal Dystonia-Associated Genes

Symbol	Gene	Locus	Mode of inheritance	No. patients	Current age /Age of onset (years)	Sex (M/F)	Laryngeal dystonia (adductor/ abductor)	Clinical presentation	Literature
**DYT1**	*TOR1A*	9q34	AD	2	48/39	F	adductor	cervical dystonia, spasmodic dysphonia (SD) and writer's cramp	Xiao *et al*. [22]
					63/19	F	adductor	SD, upper extremity action tremor	
**DYT4**	*TUBB4A*	19p13	AD	9	30-65 range /24.1 ± 7.7	5F/4M	adductor	generalized dystonia, upper limb tremor even after bilateral ventrolateral nuclei thalamotomy; subtle swallowing problems; gait dystonia	Wilcox *et al*.[23]
				2	31/20	F	adductor	whispering voice and eyelid ptosis; later on severe dysphagia requiring supplemental feeding through a percutaneous endoscopic gastrostomy (PEG) tube; “hobby horse” type gait	Lohmann *et al.* [24]
					71/60	F	adductor	SD, oromandibular dystonia and Dyskinesia	

**DYT6**	*THAP1*	8p11	AD	2	35/9	M	*not specified	severe LD, speech problems followed by writer's dystonia, dysarthria	Djarmati *et al*. [25]
					68/10	M	*not specified	writer's dystonia followed by LD	
				6	58/51	F	adductor		Xiao *et al*. [26]
					66/62	F	adductor		
					63/62	F	adductor		
					85/33	F	adductor	segmental craniocervical dystonia that included LD, blepharospasm, and lower facial dystonia	
					62/48	F	adductor		
					75/66	F	adductor		
				1	35/14	F	adductor	spasmodic dysphonia that spread to the oromandibuar region	Groen *et al*. [27]
				2	NA/14	F	adductor	SD with cervical and oromandibular spread	Groen *et al*. [28]
					35/14	F	adductor	SD that spread to the oromandibular region, tongue dystonia	

				3	24/8	M	abductor	right arm dystonia and bilateral upper extremity dystonic tremors; mild cervical, lower facial, lingual, and masticatory dystonia	LeDoux *et al.* [29]
					44/6	M	abductor	SD, blepharospasm, masticatory dystonia, lower facial dystonia, lingual dystonia, bilateral proximal arm dystonia (left > right), bilateral distal arm dystonia (right > left), truncal dystonia, and mild asymmetrical dystonia in the legs and feet	
					47/40	M	abductor	LD and bilateral dystonic arm tremors, blepharospasm and lower facial, lingual and masticatory dystonia	
				2	70/24	M	*not specified	SD	de Gusmão *et al.* [30]
					NA/50	F	*not specified	focal SD	
**DYT11**	*SGCE*	7q21	AD	1	66/40	M	*not specified	writer's cramp, myoclonus dystonia	Kumar *et al.* [31]
				1	24/4	M	*not specified	myoclonus dystonia with SD	Gultekin *et al.* [32]

**DYT16**	*PRKRA*	2q31	AR	4	35/12	M	*not specified	oromandibular dystonia, laterocollis, and upper limb dystonia, present parkinsonism	Camargos *et al.* [33]
					19/2	M	*not specified	generalised dystonia characterised by involvement of legs, hands, trunk, and voice, severe bradykinesia but no tremor	
					64/18	M	*not specified	laterocollis, right laterotrunk, SD, extension of the left leg when walking, slight bradykinesia, present parkinsonism	
					48/2	F	*not specified	combination of lower volume and strangled character suggestive of a mixed form of SD, bradykinesia, present parkinsonism	
				1	35/13	M	adductor	SD, oromandibular dystonia, and dystonic protrusion of the tongue, parkinsonism	Camargos *et al.* [34]
				1	40/39	F	*not specified	LD	Zech *et al.* [35]
				2	43/8	M	*not specified	SD, parkinsonism	Quadri *et al.* [36]
					38/4	M	*not specified	dystonia of the oromandibular region (reduced by a sensory trick - a light touch on the chin), the neck	

								(laterocollis and antecollis), and both upper limbs and right foot, parkinsonism	
**DYT24**	*ANO3*	11p14	AD	1	NA/NA	F	*not specified	cervical, laryngeal, and upper-limb tremulous dystonia	Charlesworth *et al*. [37]
				7	39/5	F	*not specified	LD and mild oromandibular dystonia, blepharospasm and torticollis, head tremor	Stamelou *et al*. [38]
					31/6	F	adductor	essential tremor	
					deceased/27	F	adductor	torticollis, head tremor	
					67/40	M	adductor	torticollis to the right, blepharospasm, head tremor	
					69/30	M	adductor	torticollis, blepharospasm, oromandibular, head tremor	
					deceased/30	F	adductor	torticollis, head tremor	
					36/19	F	adductor	torticollis, head tremor	

**DYT25**	*GNAL*	18p11	AD	1	37/36	M	adductor	LD	Putzel *et al.* [39]
				1	42/32	F	*not specified	difficulty pronouncing vowels, neck dystonia, blepharospasm and writer's cramp	Kumar *et al*. [31]
**DYT27**	*COL6A3*	2q37	AR	2	51/20	M	*not specified	tremulous cervical dystonia; upper limb dystonia with dystonic action and postural tremor; writer's cramp; oromandibular dystonia; LD; trunk dystonia	Zech *et al.* [40]
					48/20	F	*not specified	tremulous cervical dystonia; upper limb dystonia with dystonic action and postural tremor; writer's cramp; oromandibular dystonia; LD	
**[2DYT 28**	*KMT2B*	19q13	AD	19	6-46/2-23	10F/9M	*not specified		Meyer *et al.* [41]

	1	28/6	M	*not specified	oromandibular-laryngeal involvement and atypical gait dystonia (*Charlot gait*)	Brás *et al*. [42]
	11	10-52/3-13	7F/4M	abductor /adductor type	progressing to scarcely intelligible speech or even complete anarthria in 4 patients, oromandibular dystonia, parkinsonism	Carecchio *et al*. [43]
	32	3-44/3–19	18F/14M	*not specified		Cif *et al*. [44]
	1	23/5	M	*not specified	LD, generalized dystonia with a tremulous and twisting pattern	Kumar *et al.* [31]

AD, autosomal dominant; AR, autosomal recessive; SD, spasmodic dysphonia; LD, laryngeal dystonia; NA, not available; not specified, the authors did nit specfied what type of dystonia is, the addutcor or abductor type

References

[22] Xiao, J.; Bastian, R.W.; Perlmutter, J.S.; Racette, B.A.; Tabbal, S.D., Karimi M, et al. High-throughput mutational analysis of TOR1A in primary dystonia. *BMC Medical Genetics* **2009**, *10*, 24. https://doi.org/10.1186/1471-2350-10-24

[23] Wilcox, R. A.; Winkler, S.; Lohmann, K.; Klein, C. Whispering dysphonia in an Australian family (DYT4): a clinical and genetic reappraisal. *Mov Disord* **2011**, 26, 2404-2408. https://doi.org/10.1002/mds.23866

[24] Lohmann, K.; Wilcox, R. A.; Winkler, S.; Ramirez, A.; Rakovic, A.; Park, J. S.; Arns, B.; Lohnau, T.; Groen, J.; Kasten, M.; Brüggemann, N.; Hagenah, J.; Schmidt, A.; Kaiser, F. J.; Kumar, K. R.; Zschiedrich, K.; Alvarez-Fischer, D.; Altenmüller, E.; Ferbert, A.,;Lang, A. E.; Klein, C. (2013). Whispering dysphonia (DYT4 dystonia) is caused by a mutation in the TUBB4 gene. *Ann Neurol* **2013**, *73*, 537-545. 17.https://doi.org/10.1002/ana.23829

[25] Djarmati, A.; Schneider, S. A.; Lohmann, K.; Winkler, S.; Pawlack, H.; Hagenah, J.; Brüggemann, N.; Zittel, S.; Fuchs, T.; Raković, A.; Schmidt, A.; Jabusch, H. C.; Wilcox, R.; Kostić, V. S.; Siebner, H.; Altenmüller, E.; Münchau, A.; Ozelius, L. J.; Klein, C. Mutations in THAP1 (DYT6) and generalised dystonia with prominent spasmodic dysphonia: a genetic screening study. The Lancet. *Neurology* **2009**, *8*, 447–452. https://doi.org/10.1016/S1474-4422(09)70083-3

[26] Xiao, J.; Zhao, Y.; Bastian, R. W.; Perlmutter, J. S.; Racette, B. A.; Tabbal, S. D.; Karimi, M.; Paniello, R. C.; Wszolek, Z. K.; Uitti, R. J.; Van Gerpen, J. A.; Simon, D. K.; Tarsy, D.; Hedera, P.; Truong, D. D.; Frei, K. P.; Dev Batish, S.; Blitzer, A.; Pfeiffer, R. F.; Gong, S.; … LeDoux, M. S. Novel THAP1 sequence variants in primary dystonia. *Neurology* **2010**, *74*, 229–238. https://doi.org/10.1212/WNL.0b013e3181ca00ca

[27] Groen, J. L., Yildirim, E., Ritz, K., Baas, F., van Hilten, J. J., van der Meulen, F. W., Langeveld, T. P., & Tijssen, M. A. THAP1 mutations are infrequent in spasmodic dysphonia. *Mov Disord* **2011**, *26*, 1952-194. https://doi.org/10.1002/mds.23682

[28] Groen, J. L.; Kallen, M. C.; van de Warrenburg, B. P.; Speelman, J. D.; van Hilten, J. J.; Aramideh, M.; Boon, A. J.; Klein, C.; Koel-man, J. H.; Langeveld, T. P.; Baas, F.; Tijssen, M. A. Phenotypes and genetic architecture of focal primary torsion dystonia. *J Neurol Neurosurg Psychiatry* **2012**, *83*, 1006-1011. https://doi.org/10.1136/jnnp-2012-302729

[29] LeDoux, M. S.; Xiao, J.; Rudzińska, M.; Bastian, R. W.; Wszolek, Z. K.; Van Gerpen, J. A.; Puschmann, A.; Momčilović, D.; Vemula, S. R.; Zhao, Y. Genotype-phenotype correlations in THAP1 dystonia: molecular foundations and description of new cases. *Parkinsonism Relat Disord* **2012**, *18*, 414-425. https://doi.org/10.1016/j.parkreldis.2012.02.001

[30] de Gusmão, C. M.; Fuchs, T.; Moses, A.; Multhaupt-Buell, T.; Song, P. C.; Ozelius, L. J.; Franco, R. A.; Sharma, N. Dystonia-Causing Mutations as a Contribution to the Etiology of Spasmodic Dysphonia. *Otolaryngol Head Neck Surg* **2016**, *155*, 624-628. https://doi.org/10.1177/0194599816648293

[31] Kumar, K. R.; Davis, R. L.; Tchan, M. C.; Wali, G. M.; Mahant, N.; Ng, K.; Kotschet, K.; Siow, S. F.; Gu, J.; Walls, Z.; Kang, C.; Wali, G.; Levy, S.; Phua, C. S.; Yiannikas, C.; Darveniza, P.; Chang, F.; Morales-Briceño, H.; Rowe, D. B.; Drew, A.; … Sue, C. M. (2019). Whole genome sequencing for the genetic diagnosis of heterogenous dystonia phenotypes. *Parkinsonism Relat Disord* **2019**, *69*, 111-118.. https://doi.org/10.1016/j.parkreldis.2019.11.004

[32] Gultekin, M.; Prakash, N.; Ganos, C.; Mirza, M.; Bayramov, R.; Bhatia, K. P.; Mencacci, N. E. (2019). A Novel SGCE Nonsense Variant Associated With Marked Intrafamilial Variability in a Turkish Family With Myoclonus-Dystonia. *Mov Disord Clin Pract* **2019**, 6, 479-482https://doi.org/10.1002/mdc3.12805

[33] Camargos, S.; Scholz, S.; Simón-Sánchez, J.; Paisán-Ruiz, C.; Lewis, P.; Hernandez, D.; Ding, J.; Gibbs, J. R.; Cookson, M. R.; Bras, J.; Guerreiro, R.; Oliveira, C. R.; Lees, A.; Hardy, J.; Cardoso, F.; Singleton, A. B. DYT16, a novel young-onset dysto-nia-parkinsonism disorder: identification of a segregating mutation in the stress-response protein PRKRA. *Lancet Neurol* **2008**, *7*, 207-215.. https://doi.org/10.1016/S1474-4422(08)70022-X

[34] Camargos, S.; Lees, A. J.; Singleton, A.; Cardoso, F. DYT16: the original cases. *J Neurol Neurosurg Psychiatry*, **2012**, *83*, 1012-1014. https://doi.org/10.1136/jnnp-2012-302841

[35] Zech, M.; Castrop, F.; Schormair, B.; Jochim, A.; Wieland, T.; Gross, N.; Lichtner, P.; Peters, A.; Gieger, C.; Meitinger, T.; Strom, T.M.; Oexle, K.; Haslinger, B. Winkelmann, J. DYT16 revisited: Exome sequencing identifies PRKRA mutations in a European dystonia family. *Mov Disord* **2014**, *29*, 1504-1510. https://doi.org/10.1002/mds.25981

[36] Quadri, M.; Olgiati, S.; Sensi, M.; Gualandi, F.; Groppo, E.; Rispoli, V.; Graafland, J.; Breedveld, G. J.; Fabbrini, G.; Berardelli, A.; Bonifati, V. (2016). PRKRA Mutation Causing Early-Onset Generalized Dystonia-Parkinsonism (DYT16) in an Italian Family. *Mov Disord* **2016**, *31*, 765-7. https://doi.org/10.1002/mds.26583

[37] Charlesworth, G.; Plagnol, V.; Holmström, K. M.; Bras, J.; Sheerin, U. M.; Preza, E.; Rubio-Agusti, I.; Ryten, M.; Schneider, S. A.; Stamelou, M.; Trabzuni, D.; Abramov, A. Y.; Bhatia, K. P.; Wood, N. W. (2012). Mutations in ANO3 cause dominant craniocervical dystonia: ion channel implicated in pathogenesis. *Am J Hum Genet* **2012**, *91*, 1041-1050. https://doi.org/10.1016/j.ajhg.2012.10.024

[38] Stamelou, M.; Charlesworth, G.; Cordivari, C.; Schneider, S. A.; Kägi, G.; Sheerin, U. M.; Rubio-Agusti, I.; Batla, A.; Houlden, H.; Wood, N. W.; Bhatia, K. P. The phenotypic spectrum of DYT24 due to ANO3 mutations. *Mov Disord* **2014**, *29*, 928-34. https://doi.org/10.1002/mds.25802.

[39] Putzel, G. G.; Fuchs, T.; Battistella, G.; Rubien-Thomas, E.; Frucht, S. J.; Blitzer, A.; Ozelius, L. J.; Simonyan, K. GNAL mutation in isolated laryngeal dystonia. *Mov Disord* **2016**, *31*, 750-755. https://doi.org/10.1002/mds.26502

[40] Zech, M.; Lam, D. D.; Francescatto, L.; Schormair, B.; Salminen, A. V.; Jochim, A.; Wieland, T.; Lichtner, P.; Peters, A.; Gieger, C.; Lochmüller, H.; Strom, T. M.; Haslinger, B.; Katsanis, N.; Winkelmann, J. Recessive mutations in the $\alpha 3$ (VI) collagen gene COL6A3 cause early-onset isolated dystonia. *Am J Hum Genet* **2015**, *96*, 883-893. https://doi.org/10.1016/j.ajhg.2015.04.010

[41] Meyer, E.; Carss, K. J.; Rankin, J.; Nichols, J. M.; Grozeva, D.; Joseph, A. P.; Mencacci, N. E.; Papandreou, A.; Ng, J.; Barral, S.; Ngoh, A.; Ben-Pazi, H.; Willemsen, M. A.; Arkadir, D.; Barnicoat, A.; Bergman, H.; Bhate, S.; Boys, A.; Darin, N.; Foulds, N.; Kurian, M. A. (2017). Mutations in the histone methyltransferase gene KMT2B cause complex early-onset dystonia. *Nat Genet* **2017**, *49*, 223-237. https://doi.org/10.1038/ng.3740

[42] Brás, A.; Ribeiro, J. A.; Sobral, F.; Moreira, F.; Morgadinho, A.; Januário, C. Early-onset oromandibular-laryngeal dys-tonia and Charlot gait: New phenotype of DYT-KMT2B. *Neurology* **2019**, *92*, 919. https://doi.org/10.1212/WNL.0000000000007469

[43] Carecchio, M.; Invernizzi, F.; Gonzàlez-Latapi, P.; Panteghini, C.; Zorzi, G.; Romito, L.; Leuzzi, V.; Galosi, S.; Reale, C.; Zibordi, F.; Joseph, A.P.; Topf, M.; Piano, C.; Bentivoglio, A.R.; Girotti, F.; Morana, P.; Morana, B.; Kurian, M.A.; Garavaglia, B.; Mencacci, N.E.; Lubbe, S.J.; Nardocci, N. Frequency and phenotypic spectrum of KMT2B dystonia in childhood: A single-center cohort study. *Mov Disord* **2019**, *34*, 1516-1527. https://doi.org/10.1002/mds.27771

[44] Cif, L.; Demailly, D.; Lin, J. P.; Barwick, K. E.; Sa, M.; Abela, L.; Malhotra, S.; Chong, W. K.; Steel, D.; Sanchis-Juan, A.; Ngoh, A.; Trump, N.; Meyer, E.; Vasques, X.; Rankin, J.; Allain, M. W.; Applegate, C. D.; Attaripour Isfahani, S.; Baleine, J.; Balint, B.; … Kurian, M. A. KMT2B-related disorders: expansion of the phenotypic spectrum and long-term efficacy of deep brain stimulation. Brain 2020, 143, 3242-3261. https://doi.org/10.1093/brain/awaa304
